# Integration of organic–inorganic nitrogen fertilization on nitrogen conversion in soil

**DOI:** 10.3389/fpls.2025.1688878

**Published:** 2025-12-10

**Authors:** Akhlaq Ahmad, Aaqil Khan, Farhan Ullah, Muhammad Ahmad Hassan, Javed Iqbal, Wang Jun, Song He, Dong Zhaorong

**Affiliations:** 1 College of Agronomy, Anhui Agricultural University, Hefei, China; 2 College of Coastal Agriculture Sciences, Guangdong Ocean University, Zhanjiang, China; 3 National Engineering Laboratory of Crop Stress Resistance Breeding, School of Life Sciences, Anhui Agricultural University, Hefei, China

**Keywords:** nitrogen fertilization, microbial dynamics, soil health, gene expression, nutrient cycling

## Abstract

The excessive use of inorganic nitrogen fertilizers poses significant environmental threats, including nitrate leaching and greenhouse gas emissions. Conversely, organic fertilizers enhance soil microbial activity and long-term fertility. This study investigates the integrated application of organic and inorganic nitrogen fertilizers to optimize soil health and crop productivity while mitigating environmental impacts. A field experiment was conducted over a single maize growing season using a randomized block design. Six treatments were applied: no fertilizer (T1), chemical fertilizer only (T2), 15% (T3), 30% (T4), or 45% (T5) sheep manure combined with chemical fertilizer, and sheep manure only (T6). Soil samples were analyzed for pH, dissolved organic carbon (DOC), nitrate (NO₃⁻), ammonium (NH₄⁺), and the microbial gene abundance of key nitrifiers (*AOA*, *AOB*) and denitrifiers (*nirK*, *nirS*). The 100% sheep manure treatment (T6) resulted in significantly higher soil DOC (34 ± 4.58 mg/kg) and NO₃⁻ (3.31 ± 0.51 mg/kg) concentrations, promoting enhanced microbial diversity and activity. In contrast, the inorganic-only treatment (T2) yielded the highest grain nitrogen content (9.43 ± 2.24 mg/kg), indicating immediate nutrient availability. Heat map and Principal Component Analysis (PCA) revealed distinct clustering of microbial gene expressions (*AOA*, *AOB*, *nirK*, *nirS*) in response to fertilization, with high organic inputs fostering a more diverse and active microbial community. Redundancy Analysis (RDA) showed strong correlations between these environmental factors and gene expression. The results demonstrate that integrating organic and inorganic fertilizers creates a synergistic effect. This approach optimizes nutrient cycling by balancing immediate crop availability with long-term soil building. It enhances microbial functional capacity for nitrification and denitrification, thereby improving soil health and reducing potential environmental impacts like nitrate leaching and greenhouse gas emissions. We conclude that the integration of organic and inorganic fertilization is a key strategy for supporting sustainable agricultural productivity.

## 1 Introduction

Nitrogen is vital in agricultural systems as it is a fundamental macronutrient needed for the synthesis of amino acids, proteins, and nucleic acids, which are essential for the growth of plants (Raina and Mazahar, 2022; Hawkesford et al., 2023). The modern application of inorganic nitrogen fertilizers has substantially improved the yields of crops, but it has also caused various environmental issues (Sainju et al., 2019; Ilahi et al., 2020; Tyagi et al., 2022). These include nitrate leaching, leading to groundwater and surface water contamination and the emission of nitrous oxide, which is a powerful greenhouse gas (Zhang et al., 2020; Rath et al., 2021; Bouhout et al., 2024). The continuous use of inorganic nitrogen fertilizers results in high production cost and nitrate runoff, which has led to the observed environmental problems. Similarly, nitrate leaching results in groundwater pollution, thus causing health-related problems to human beings upon consumption (Wang and Li, 2019; Min et al., 2021). Acidification of soils as a result of the excessive use of inorganic nitrogenous fertilizers also affects soil health, as the soils do not have the desired microbial communities and nutrients (Hao et al., 2020; Raza et al., 2021; Ye et al., 2023).

Moreover, the inadequate or excessive application of inorganic nitrogenous fertilizers adversely affect soil health, as soil nutrient availability will be interfered (Baweja et al., 2020; Bangre et al., 2024). The noted environmental problems call for proper nitrogen management, one that will ensure that a high-yield agriculture balances with environmental conservation (Tei et al., 2020; Mahmud et al., 2021). In spite of these, the integrated organic and inorganic nitrogen fertilization is becoming a better alternative due to the benefits of organic nitrogen sources (Walling and Vaneeckhaute, 2020; Harindintwali et al., 2021). Organic nitrogen sources such as manure and compost are associated with a slow release of nutrients (Shaji et al., 2021). Soil organic matter accumulation is improved, and so is the soil microbial activity (Shao et al., 2019; Bhattacharyya et al., 2022). Additionally, the organic sources of nitrogen also benefit the users in that nutrient cycles are enhanced over the long term, and these keep the soils fertile (Ashworth et al., 2020; Silveira and Kohmann, 2020).

On the other hand, it can be stated that inorganic fertilizers offer an instant nutrient supply which is impossible to meet with the help of the other sources required by the demands of rapid growth of crops (Moe et al., 2019; Pahalvi et al., 2021). In such a way, the only usage of inorganic fertilizers will lead to a low rate of nutrient proper balance as well as decreased soil organic matter, and such chain reactions will define the decrease in soil fertility and the increase in environmental pollution (Hammad et al., 2020; Yang et al., 2020). The ultimate decision is to combine these two types to reach optimal performance in nutrient availability, soil structure changes, and agricultural system development.

Previous research has demonstrated that integrated organic–inorganic fertilization enhances nutrient use efficiency and reduces the environmental footprint of agriculture (Sileshi et al., 2019; Verma et al., 2019; Rashmi et al., 2020; Bhunia et al., 2021; Oyetunji et al., 2022). These benefits are mediated by improved soil microbial biomass and activity, which promote nutrient mineralization and availability (Ashraf et al., 2021). However, the efficacy of this approach is influenced by site-specific factors such as soil properties, crop type, and ecological conditions (Celestina et al., 2019; Techen et al., 2020).

Ammonia-oxidizing archaea *AOA* and ammonia-oxidizing bacteria *AOB* have a significant impact in the nitrification process (Rütting et al., 2021; Zou et al., 2022). Nitrification is one of the nitrogen cycle transformation steps (Robertson and Groffman, 2024). It can be facilitated by the detection of the presence and abundances of these organisms based on their expression of certain specific genes (Hoang et al., 2022). Both organisms have related forms of the gene, and its variants are biomarkers used in studying nitrification in soils (Guo et al., 2021; Lu et al., 2022). However, applying the substances rich in ammonia initially has a larger contribution to *AOB* activation because of the direct use of its substrate (Sun et al., 2021; Yang et al., 2023). The application of organic amendments like sheep manure can alter ammonia-oxidizing community dynamics by introducing substrates and compounds that stimulate or inhibit ammonia-oxidizing bacteria (*AOB*) (Coelho, 2019; He et al., 2024). This effect is contingent on the manure’s composition, particularly its ammonia concentration and carbon sources (Wu et al., 2022; Tebaldo et al., 2024). It is suggested by previous research that higher application rates often stimulate ammonia-oxidizing archaea (*AOA*) abundance, which is attributed to their adaptation to organic-matter-rich, ammonia-limiting environments (Lin et al., 2021; Xiao R. et al., 2021; Yao et al., 2022; Basak et al., 2023).

*nirK* and *nirS* are types of genes that encode nitrite reductase enzymes that are necessary in the denitrification process (Wittorf et al., 2018; Ye et al., 2021). Denitrification is mediated by microbes and the reduction of nitrate to forms of gaseous nitrogen such as nitrous oxide (Shan et al., 2021; LaSarre et al., 2024). In soil, the expression ability of these genes implies the denitrification potential of the microbial communities and their ability to prevent the accumulation of nitrate, thereby reducing the risk of contaminating the environment with nitrate leaching and N_2_O emissions (Kumar et al., 2020; Shakywal et al., 2023). The use of organic nitrogenous sources enhances *nirK* and *nirS* gene overloads especially when used in conjunction with inorganic nitrogen sources (Arumugham et al., 2024; Fan et al., 2024). These are likely to be enhanced because of the availability of excess organic carbon, which serves as an electron donor (Pang and Wang, 2021). Conversely, the expression over-loads are likely to be reduced due to inorganic N fertilizer overloads (Dimkpa et al., 2023). In such an event, the production of N_2_O could increase since the expression of denitrification is likely to be reduced (Duan et al., 2018).

The optimum levels of soil pH with the concentration of nitrate (NO_3−_) and ammonium (NH_4_^+^) are essential indicators of soil health and fertility (Sahoo et al., 2022). Soil pH affects the availability of nutritional substances in soil, behavior of microbes, and general soil chemistry (Barrow and Hartemink, 2023). The overall action is to balance the addition of both manure and inorganic fertilizers to maintain the optimum pH range that supports higher microbial diversity, thereby fostering soil support (Philippot et al., 2024).

Unlike inorganic fertilizers that rapidly acidify soil and raise nitrate levels increasing volatility and contamination risk (Akhtar et al., 2021; Lazcano et al., 2021), organic amendments (sheep manure) buffer pH and mitigate nitrate leaching (Goldan et al., 2023; Shakywal et al., 2023). Excessive inorganic fertilizers degrade soil and cause pollution, while organic manure overuse can lead to nutrient surpluses and greenhouse gas emissions (Zhang et al., 2012; Liu et al., 2020; Fan et al., 2022).

As much as carbon dioxide and nitrous oxide emissions are a cause of serious alarm, it is also an important factor indicating the effects of artificially and naturally produced fertilizers on the environment (Benghzial et al., 2023; Nunes, 2023). The total amount of CO_2_ emitted in agriculture results from soil respiration and other microbial activities plus the influence of organic and inorganic nitrogen additions (Almagro et al., 2023). Nitrogen, a product of nitrification and denitrification, is the precursor of N_2_O gas (Chalk and Smith, 2021). The application of organic nitrogen fertilizers often leads to a buildup of partially reduced nitrogen forms due to microbial competition, but the impact of this and how to manage it vary with soil conditions (Stavi et al., 2021). In light of the stated implications, the integrated application of organic (including sheep manure) and inorganic nitrogen fertilizers is a promising trend for sustainable soil management (Iqbal et al., 2019); however, it requires careful balancing to maximize benefits and minimize the risk of the ongoing challenges including nutrient management, manure quality variability, and environmental concerns (Liu et al., 2020; Liu et al., 2021).

To ascertain the management complexity, variability in feralization quality, and effects of nutrient imbalance and pollution (Liu et al., 2020; Fan et al., 2022), the objectives of the present study are stated as follows: (1) to evaluate the combined effect of organic (sheep manure) and inorganic nitrogen fertilization on soil nitrogen dynamics and greenhouse gas emissions and (2) to assess the influence on these fertilization strategies on key soil health indicators, including pH, nitrate (NO_3−_) and ammonium (NH_4_^+^) concentrations, and microbial activity. We hypothesized that the integration of organic and inorganic nitrogen sources would enhance soil health and crop productivity while reducing negative environmental impacts such as nitrate leaching and greenhouse gas emissions. To test this hypothesis, we conducted this experiment comparing various proportions of organic and inorganic nitrogen treatments. The integrated approach of the study involved using different analytical methods to determine the multiple implications of fertilization strategies on soil condition and harvest results. First, independent samples *t*-tests and ANOVA were planned to statistically assess the differences between various treatments based on multiple factors related to soil and crops. Second, following the study designs, the soil microbial community structures and functions were analyzed with the help of pyrosequencing and real-time PCR, with special attention to the relevant genes for ammonia oxidation that include associated ammonia monooxygenase *AOA* and *AOB* genes as well as the *nirK* and *nirS* genes. The chosen genes were considered to be crucial in estimating the soil’s nitrification and denitrification potential under different types of fertilization. The integrated approach was chosen based on multiple goals related to the support of sustainable development of agricultural practices and the promotion of efficient nutrient cycling with a focus on optimal harvest results and soil fertility.

## 2 Materials and methodology

### 2.1 Experimental site

A field plot experiment was performed during 2019–2020 in North Wanbei Complex Experimental Station of Anhui Agricultural University, located in Fuhu Village, Huigu Town, Yongqiao District, Suzhou City, Anhui Province of China (33°41′ N, 117°04′ E). The experimental site is located at the southern end of the Huanghuai Plain. The climate of this area is warm temperate, semi-humid, and with monsoon. The average sunshine duration is 2,350 h, the annual average temperature is 15.7°C, the frost-free period is about 210 days, and the annual average precipitation is 857 mm. The soil at the test site is Shajiang black soil, an alkaline, sticky, and heavy-textured type, typical of the southern Huanghuai Plain. Based on its organic matter content, phosphorus (P), and potassium (K) levels, it is classified as Chernozem (FAO).

### 2.2 Experimental design and treatments

A completely randomized design block was used, involving six treatments replicated three times across plots measuring 60 m^2^ (6 ^m^ × 10 m). Sheep manure was taken as the organic source, and urea fertilizer was taken as the chemical fertilizer. Sheep dung was collected from the farm. The sheep dung was mixed with soil 10 days before planting. The control pots received no fertilizer and sheep dung. The treatments were T1: control (no fertilization), T2: inorganic fertilizer only, T3: inorganic fertilizer + sheep manure 15%, T4: inorganic fertilizer + sheep manure 30%, T5: inorganic fertilizer + sheep manure 45%, and T6: sheep manure 100%. Each fertilization treatment adopts the consistent experimental plan of nitrogen, phosphorus, and potassium, which are nitrogen fertilizer (N): 240 kg·hm^-2^, phosphate fertilizer (P_2_O_5_): 120 kg·hm^-2^, and potassium (K_2_O): 240 kg·hm^-2^ (**Supplementary Table 1**). Conventional chemical fertilizers were used for the application of chemical fertilizers: ammonium diphosphate (N: 18%–P_2_O_5_: 46%), urea (46.4% N), and potassium sulfate (50% K_2_O). The moisture content, organic matter content, total nitrogen, total phosphorus, and total potassium of sheep manure in corn seasons were 42.1% and 16.83, 2.27, 0.941, and 3.77 g·kg^-1^. The corn variety was Zhengdan 958. The organic fertilizer used in the experiment was fermented and corroded sheep manure. The sowing method was machine drill sowing. In the early stage of planting, sheep manure and some chemical fertilizers were used as base fertilizer. The remaining nitrogen fertilizer is used in the trumpet stage of corn (base fertilizer: top dressing = 7:3). The planting density of corn was 55,500 plants·hm^-2^, and the sowing date is June 18, 2019.

### 2.3 Soil sampling and chemical analysis

Soil was taken from the 20-cm-deep layer of the experimental site before the harvest of maize. The soil was crushed by hand into smaller particles and air-dried, and then the soil was passed through a 2-mm sieve to remove the rock coarse, plant materials, and other contaminations. The soil samples were analyzed to determine their chemical and physical properties. For pH determination, the soil samples were mixed with deionized water at 1:5 soil-to-water ratios with the help of a glass stirrer. The pH of the soil samples was measured by using the pH meter. Electrical conductance of the soil was valued by mixing the soil sample with deionized water and then storing it at room temperature for 24 h. The electrical conductance of the mixture was measured with the help of an electrical conductance meter. The concentration of soil dissolved organic carbon was extracted by 0.5 M K_2_SO_4_ solutions, and it was analyzed with multi 3100 C/N analyzer. The level of organic matter in soil was determined by external heating with the potassium dichromate volumetric method (Gerenfes et al., 2022). The total nitrogen (N) content in the soil was determined using a carbon and nitrogen analyzer (Vario MACRO CN, Germany). Ammonium nitrogen (NH_4_^+^–N) and nitrate nitrogen (NO_3−_–N) were extracted in 1.0 M KCl, respectively, and analyzed on a continuous-flow injection analyzer (TRAACS2000, Hamburg, Germany).

#### 2.3.1 Basal soil respiration

The calculation of basal soil respiration was according to (Pell et al., 2006). A total of 20 g of soil was amended to 60% water-holding capacity and transferred into a 250-mL respirometry jar. During a period of 7 days, the jars were incubated in a Respicond III respirometer (Nordgren Innovation AB, Umeå) at 22°C. Production of CO_2_ was collected in a 0.2-M potassium hydroxide solution (10 mL), and the conductivity of the solution was recorded at an interval of 30 min to calculate respiration per unit time.

#### 2.3.2 Plant nitrogen content

The samples were taken at the harvest stage from each plot to determine the nitrogen uptake in plants. The samples were divided in stems and leaves. Then, these samples were dried at 85 °C in an oven. Total nitrogen content was determined by using the micro Kjeldahl assay.

#### 2.3.3 Nitrification potential

The concentrated NO_2_ was extracted from the soil sample by adding 5 g of soil in 50 mL 2 M KCl solution and stirred at 250 rpm for 30 min on an orbital shaker. The samples were filtered through a Whatman filter paper and analyzed using continuous flow injection analyzer (Bundy et al., 1994).

#### 2.3.4 Denitrification potential

Fresh soil samples were collected from each sample and placed in a serum vial containing 1.5 mM NH_4_ solution in a quantity of 100 mL. Acetylene in a concentration of 0.025% was immediately added to the serum vial and sealed with rubber septa and covered with aluminum foil. Two replicates were made for each sample. After 6 h, acetylene was removed and the rubber septa were also loosened to allow the air to pass. Replicates were labeled as RNP total and RNP*_AOA_* (Taylor et al., 2012). The concentration of N was measured after 6, 24, 36, and 48 h of incubation. RNP*_AOB_* was measured by using the following equation:


$$RNP_{AOB}=RNP_{total}–RNP_{AOA}$$

#### 2.3.5 Soil greenhouse gas measurement and sampling

The sum of CH_4_ and N_2_O was measured as a static dark chamber coupled with gas chromatography (GC) to determine soil greenhouse gas emissions. The sampling system was composed of two parts: an upper box and a lower chamber. A top transparent box with dimensions of 50 cm × 50 cm × 0.5 cm was used. A cavity based on a 2-in. (5 cm) spacer system and insulated with 3/4-in. foam was intended to capture gases. The lower part was constructed in the form of a square chamber (50 × 50 × 10 cm) and gets half-embedded into the ground soil by up to at least approximately one-third out of the surface, also known as flush fit. Two ports were added along the chamber side with an access to both a three-way valve and electronic thermometer (JM22L). Gas sampling was performed between 8:30 and 11:00 a.m.

Water was added to the lower chamber prior to gas collection in each batch. Afterward, the top box is placed to cover this one with an airtight action. A syringe was used to draw 40 mL of mixed gas samples at time intervals of 0, 7, 14, and 21 min. The gas chromatograph used for the analysis is Agilent 7890A, USA. In the growth period, hypertrophic scars were sampled once a week, while in the winter it was repeated every 2 weeks. The sampling frequency was doubled during fertilization, irrigation, and intense rains. Soil temperature and moisture levels were measured by a soil water content–temperature conductivity instrument (MG-EM50, China) at the same time.

The formula to calculate the flux of N_2_O, CO_2_, and CH_4_ emissions is as follows:


$$F\;=\;\rho \;\times h\;\times \left(\frac{dc}{dt}\right)\times \frac{273}{273+T}$$

where

- F represents the emission flux of N_2_O (μg·(m²·h)^-1^)
- ρ = the gas density at standard conditions (kg·m^-3^)
- h is the net height range of sampling box (m)
- where dc/dt is the slope of gas concentration in pharmacokinetic units (μL·(L·h)-1)
- where 273 is a constant from the ideal gas law
- where T is the average ambient temperature inside our sampling chamber (°C)

The formula for the calculation of N_2_O emission factor (EF) is as follows:


$$EF=\;\frac{Amount\;of\;nitrogen\;lost\;as\;N^{2}O\;}{Amount\;of\;nitrogen\;fertilizer\;applied}$$

The concentration of CO_2_ was analyzed on gas chromatography (Agilent 7890A, Agilent Technologies, USA) equipped with a thermal conductivity detector (TCD) to measure CO_2_.

#### 2.3.6 DNA extraction and real-time PCR assay

The soil sample of 0.5 g was used to extract the DNA of soil by using FastDNA Spin Kit. The quality of DNA was determined by agarose gel electrophoresis. Then, the sample DNA was stored at 20°C for further molecular analysis and PCR. Quantitative PCR method was used to find out the abundance of *nirS* and *nirK* genes. FIaCu\R3Cu and cd3AF\R3cd were used for the amplification of these *nirS* and *nirK* genes. The real-time PCR for *nirS* was initiated at 95°C for 5 min. Similarly, *nirK* PCR was initiated at 98°C for 5 min. PCR was repeated three times for each sample. High-throughput sequencing was performed on the Illumina MiSeq platform, and bioinformatics analysis was conducted to identify operational taxonomic units (OTUs).

#### 2.3.7 High-throughput sequencing and bioinformatics analysis

The PCR copies of DNA were purified by using QIA quick gel extraction kit. Then, all of the sequences were sequenced at illumine Miseq platform (San Diego, CA, USA). Raw sequences were filtered using FLASH software and MOTHUR program. Low-quality sequences and chimeras were removed, and sequences with 97% similarities were categorized in the same operation taxonomic unit. The operation taxonomic unit sequences were compared with the sequences which have already been reported in the National Center for Biotechnology Information (NCBI). The phylogenetic studies of the *nirS* and *nirK* genes were carried out using neighbor-joining method and MEGA 5.1 software.

### 2.4 Statistical analysis

Statistical analyses and correlation were performed using SPSS 25. Redundancy analysis (RDA) and principal component analysis (PCA) were conducted using CANOCO 4.5 to explore the relationships between environmental variables and gene expression profile.

## 3 Results

### 3.1 Soil chemical characteristics

**Table 1** explores the analysis of soil parameters and shows the profound differences across different fertilization treatments. Soil pH ranges from 7.12 to 7.18 and did not fluctuate heavily, with minor acidification present in T6 (7.12 ± 0.10). Soil EC gradually increases from T1 at 0.45 ± 0.03 dS/m to T6 at 0.65 ± 0.08 dS/m PM, reflecting the increased impact of organic matter in the soil. Soil DOC is statistically higher in T6 at 34 ± 4.58 mg/kg, which is consistent with increased microbial life and decomposition of organic matter. Organic matter content goes on increasing from T1 (control) up to T6 (100% sheep manure). T6 had the highest organic matter content (24.18 ± 1.01 g·kg^−1^) among all treatments, which also showed a highly significant effect of organic inputs on soil organic matter, and the presence of sheep manure increased it to a greater extent than in other organic and inorganic combinations. Soil respiration, reflecting microbial activity, varied significantly among treatments, with the lowest in T1 (17.25 ± 0.63 mg CO_2_/kg/day) and the highest in T6 (31.83 ± 4.19 mg CO^2^/kg/day).

**Table 1:** Soil characteristics under different organic and inorganic nitrogen fertilization treatments.

Treatment	Soil pH	Soil EC	DOC (mg/kg)	Soil OM (g/kg)	Soil respiration
T1	7.14 ± 0.06 a	0.45 ± 0.03 f	23.5 ± 5.67 d	16.5 ± 0.47d	17.25 ± 0.63e
T2	7.16 ± 0.04 a	0.47 ± 0.04 e	25 ± 5.26 cd	17.65 ± .45d	19.51 ± 2.83 d
T3	7.16 ± 0.08 a	0.52 ± 0.05 d	26.5 ± 4.82 bc	18.2 ± 1.45c	23.24 ± 3.81 c
T4	7.18 ± 0.03 a	0.55 ± 0.06 c	28 ± 3.12 b	19.61 ± 1.1c	24.24 ± 4.61 bc
T5	7.14 ± 0.06 a	0.60 ± 0.07 b	30.5 ± 5.26 ab	21.62 ± 0.9b	27.69 ± 1.42 ab
T6	7.12 ± 0.1 a	0.65 ± 0.08 a	34 ± 4.58 a	24.18 ± 1.01a	31.83 ± 4.19 a

Different lowercase letters indicates statistically significant differences between the treatment means for each parameter. Values are means ± standard deviation. All values means significant difference at *p*<0.05. Here, DOC (mg/kg): Dissolved Organic Carbon (milligram/kilogram), Soil OM (g/kg): Soil Organic Matter (gram/ kilogram).

### 3.2 Effect of different nitrogen fertilization strategies on soil nitrogen dynamics

**Figure 1:**
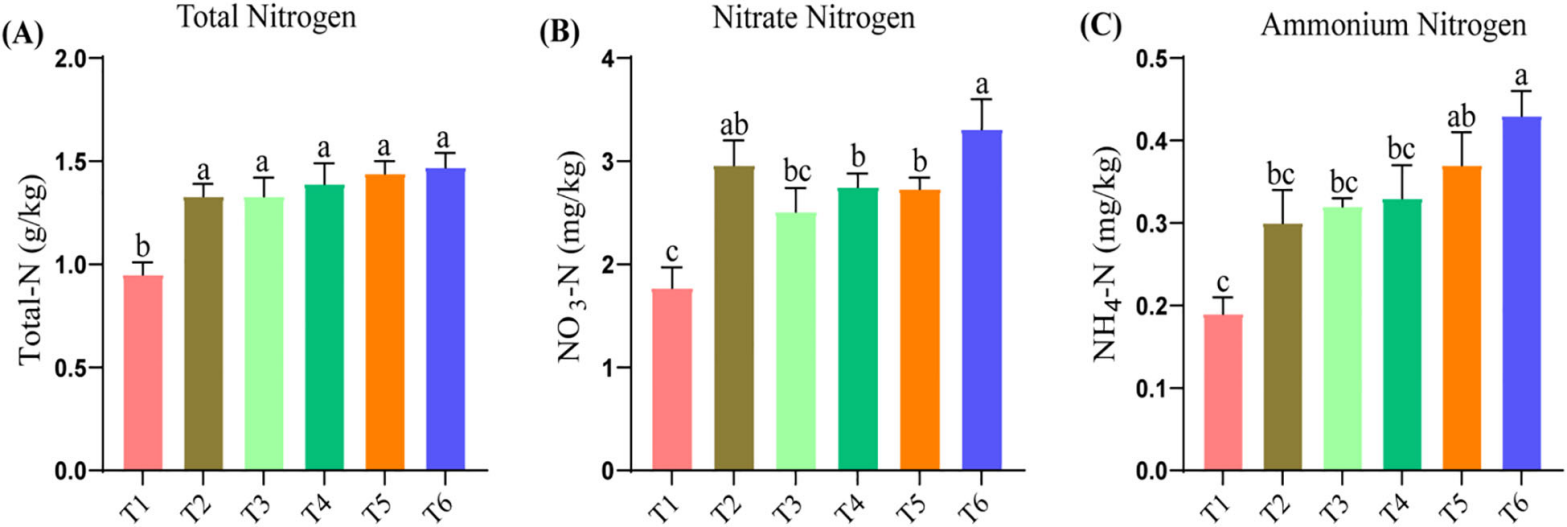
Soil nitrogen dynamics under varying levels of organic and inorganic nitrogen fertilization. Total nitrogen **(A)**, Nitrate nitrogen **(B)**, Ammonium nitrogen **(C)**. Different letters within the same columns denote statistically significant difference at p>0.05.

**Figure 2:**
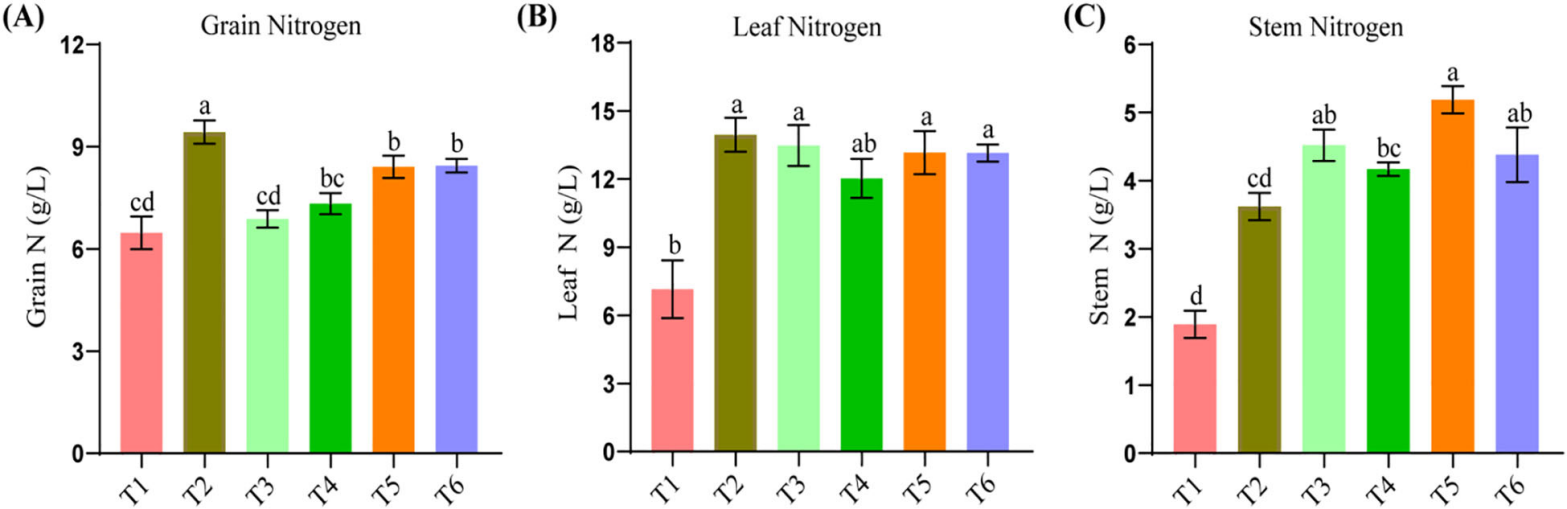
Plant nitrogen dynamics under varying organic levels of organic and inorganic nitrogen fertilization. Grain nitrogen **(A)**, Leaf nitrogen **(B)**, Stem nitrogen **(C)**. Different letters within the same columns denote statistically significant difference at p>0.05.

The total nitrogen content presents a small variation under various treatments, and the differences are not significant because it shows numerical similarity in all of the treatments from T2 through T6 (**Figure 1A**). NO_3_–N is highest in T6 at 3.31 ± 0.51 mg/kg, reflecting increased nitrification which is statistically at par with T2 (**Figure 1B**). NH_4_–N remains stable at approximately 0.30 ± 0.03 mg/kg, with the lowest found in T1 at 0.19 ± 0.02 mg/kg and the highest in T6 at 0.43 ± 0.03 mg/kg, followed by T5 (**Figure 2C**). The statistical analysis showed that the total N concentration remained fairly invariable across all of the treatments except the control, while NO_3_–N and NH_4_–N varied with fertilization differences, suggesting its impact on microbial structure and abundance.

### 3.3 Effect of different nitrogen fertilization strategies on plant nitrogen dynamics

Nitrogen content in plants is highly affected, and grain nitrogen is lowest in T1 at 6.47 ± 1.78 and highest in T2 at 9.43 ± 2.24 (**Figure 2A**). Nitrogen in leaf is not responsive to the type of fertilization, although the highest value 13.95± 1.75 was recorded at plots amended with inorganic nitrogen fertilization, yet it was statistically at par with the rest of the treatments except control (**Figure 2**). Regarding stem N, T5 resulted in higher values 5.19 ± 1.3, followed by T6 and T3 (**Figure 2C**). T2 showed the lowest values, suggesting that stem nitrogen content is more responsive to the combined application of organic and inorganic nitrogen fertilizers. The considerations above showcase the impact of fertilization strategies on plant health.

### 3.4 Nitrification potential and recovered nitrification potential analysis

**Table 2** presents the results of the nitrification potential and recovered nitrification potential analysis. There is an important difference depending on the type of fertilization, with large variability in both NP and RNP as shown by these data. Nitrification potential was highest for T2 (inorganic fertilizer) at 0.45 ± 0.04 mg N kg^−1^day^−1^, significantly different from the control at T1 (0.16 ± 0.02 mg N kg^−1^day^−1^). This demonstrates the pronounced effect of inorganic fertilizer application on nitrification potential. T2 also showed the highest RNP value of 0.23 ± 0.03 mg N kg^−1^day^−1^, and nitrification potential has recovered again at its fastest in a treatment with inorganic fertilizer application as well. However, the role of organic amendments in RNP is also prominent and showed an increasing trend as the organic fertilizer increases. By contrast, the RNP value in the control treatment was lowest, indicating least recovery potential without fertilization. T2 has been shown to be higher than other levels of RNP*AOA* and RNP*AOB* in contributions to nitrification potential; this value was 0.09 ± 0.02 mg N kg^−1^day^−1^and 0.14 ± 0.02 mg N kg^−1^day^−1^, respectively. The higher value of RNP*AOB* suggests *AOB* fascination to inorganic nitrogen fertilization as compared to *AOA*. Values of RNP*AOA* and the same reached minimum under control treatment, showing that there was minimal activity from both *AOA* and *AOB* in unfertilized soil. The result strongly indicated that inorganic and inorganic fertilizers could alter the nitrification potential, which can boost both *AOA* and *AOB* abundance and activity in soil, yet clear variances between treatments also highlight that the complement of mineral and organic input can lead to differences in degree of efficiency.

**Table 2:** Nitrification potential (NP), recovery nitrification potential (RNP) under different nitrogen fertilization treatments.

Treatment	NP (mg N kg^−1^day^−1^)	RNP (mg N kg^−1^day^−1^)	RNP*AOA* (mg N kg^−1^day^−1^)	RNP*AOB* (mg N kg^−1^day^−1^)
T1	0.16 ± 0.02 e	0.05 ± 0.01 c	0.02 ± 0.01 d	0.03 ± 0.01 d
T2	0.45 ± 0.04 a	0.23 ± 0.03 a	0.09 ± 0.02 b	0.14 ± 0.02 a
T3	0.37 ± 0.04 b	0.19 ± 0.02 b	0.07 ± 0.01 c	0.13 ± 0.01 a
T4	0.35 ± 0.03 bc	0.18 ± 0.02 b	0.09 ± 0.01 b	0.09 ± 0.01 b
T5	0.32 ± 0.02 c	0.18 ± 0.02 b	0.10 ± 0.01b	0.08 ± 0.01 bc
T6	0.30 ± 0.03 d	0.22 ± 0.02 a	0.12 ± 0.01 a	0.10 ± 0.01 b

Values are means ± standard deviation based on three replicates. Different letters within the same columns denote statistically significant difference at *p* > 0.05.

### 3.5 Denitrification potential and recovered denitrification potential analysis

Both the denitrification potential and RDP data of the different fertilization treatments exhibited a high level of variation as portrayed in **Table 3**. The DP data showed that the soil in the T6 treatment had the highest potential. This is in line with earlier results that showed that the soil was already going through a lot of degradation, and degradation involving organic manure pushes the soil’s denitrification aptitude to incredibly high levels. The potential for the control treatment T1 was low, indicating that no process was taking place and the oxidation course was going on. The DP value for the T6 treatment, 2.8 ± 0.35 mg N kg^−1^day^−1^, was significantly different and higher than that of the control treatment which is 1.5 ± 0.20 mg N kg^−1^day^−1^. This shows that the use of organic manure aids in maintaining an incredibly high denitrification potential. The RDP data resulted in higher values of 2.0 ± 0.15 at T2, highlighting the effect of inorganic nitrogen fertilization. This treatment also exhibits the highest recovery involving gene contributions to the process, the RDP *nirK* estimated at 1.4 ± 0.08 mg N kg^−1^day^−1^and the DP *nirS* estimated at 0.6 ± 0.07 x mg N kg^−1^day^−1^. This shows that the two genes have high participatory measures on the process of denitrification. The control treatment showed the lowest, T1, for both RDP *nirS* and RDP *nirK*, showing that they were inactive. In conclusion, the addition of organic properties to the soil has shown that it enhances the microbial activity of the soil, thus an increase in the denitrification process which is required for the soil to reduce its levels of nitrate and nitrous. As such, organic inputs are an integral part of the fertilization process throughout the world.

**Table 3:** Denitrification potential (DNP), recovery denitrification potential (RDP) under different nitrogen fertilization treatments.

Treatment	DP (mg N kg^−1^day^−1^)	RDP (mg N kg^−1^day^−1^)	RDP nirS (mg N kg^−1^day^−1^)	RDP nirK (mg N kg^−1^day^−1^)
T1	1.5 ± 0.20 e	0.7 ± 0.10 d	0.4 ± 0.05 d	0.3 ± 0.05 e
T2	2.1 ± 0.25 b	2.0 ± 0.15 a	0.6 ± 0.07c	1.4 ± 0.08 a
T3	2.0 ± 0.23 c	1.8 ± 0.14 b	0.8 ± 0.06 b	1.04 ± 0.07 b
T4	2.2 ± 0.28 b	1.1 ± 0.16 c	0.6 ± 0.08 c	0.5 ± 0.08 d
T5	2.5 ± 0.30 a	1.5 ± 0.20bc	0.8 ± 0.09 b	0.7 ± 0.11 c
T6	2.8 ± 0.35 a	1.8 ± 0.25 b	1.2 ± 0.10 a	0.4 ± 0.10 d

Values are means ± standard deviation based on three replicates. Different letters within the same columns denote statistically significant difference at *p* > 0.05.

### 3.6 GHG emissions from soil under different nitrogen fertilization treatments

**Figure 3:**
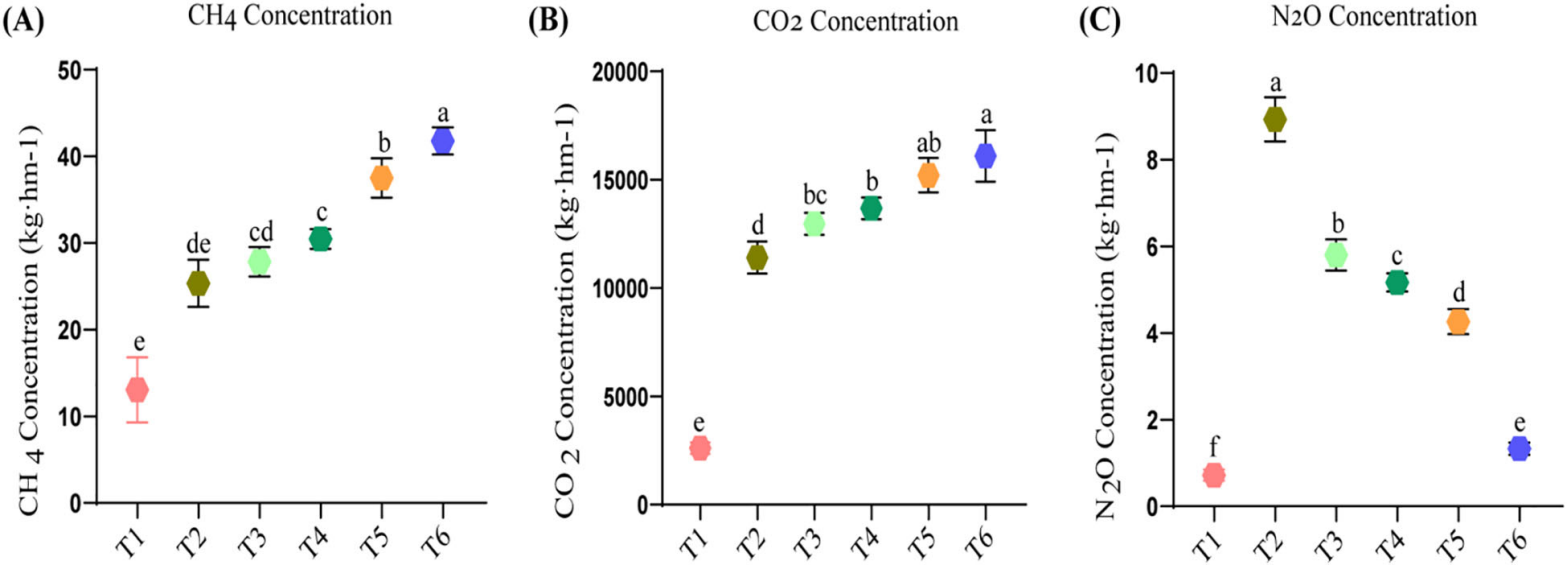
GHG Emission from soil treated with varying levels of organic and inorganic nitrogen fertilization. Methane (CH_4_) **(A)**, Carbon Dioxide (CO_2_) **(B)**, Nitrous Oxide **(C)**. Different letters within the same columns denote statistically significant difference at p>0.05.

Emissions of CH_4_, CO_2_, and N_2_O among the fertilization treatments in maize production are shown in **Figure 3**. T6 (100% sheep manure) exhibited the highest CH_4_ emissions at a rate of 41.78 ± 1.57 kg·ha^−1^, demonstrating a significantly greater contribution to methane (CH_4_) release compared to other treatments followed by T5, T4, T3, and T2 (**Figure 3A**).

Treatment T6 (100% sheep manure) also produced the highest CO_2_ emissions among all treatments, consistent with enhanced microbial activity and accelerated organic matter decomposition. In contrast, the lowest emissions were observed in T1, followed by T2 (**Figure 3B**).

The maximum N_2_O emission was recorded in T2 (100% inorganic fertilizer) which was 8.93 ± 0.51 kg·hm^−1^, and the minimum one was found in the treatments of T1 (control) as well as T6 (100% sheep manure), which indicated a notable reduction using organic inputs (**Figure 3C**).

### 3.7 Genes’ abundance across different organic and inorganic N fertilization treatments

**Figure 4:**
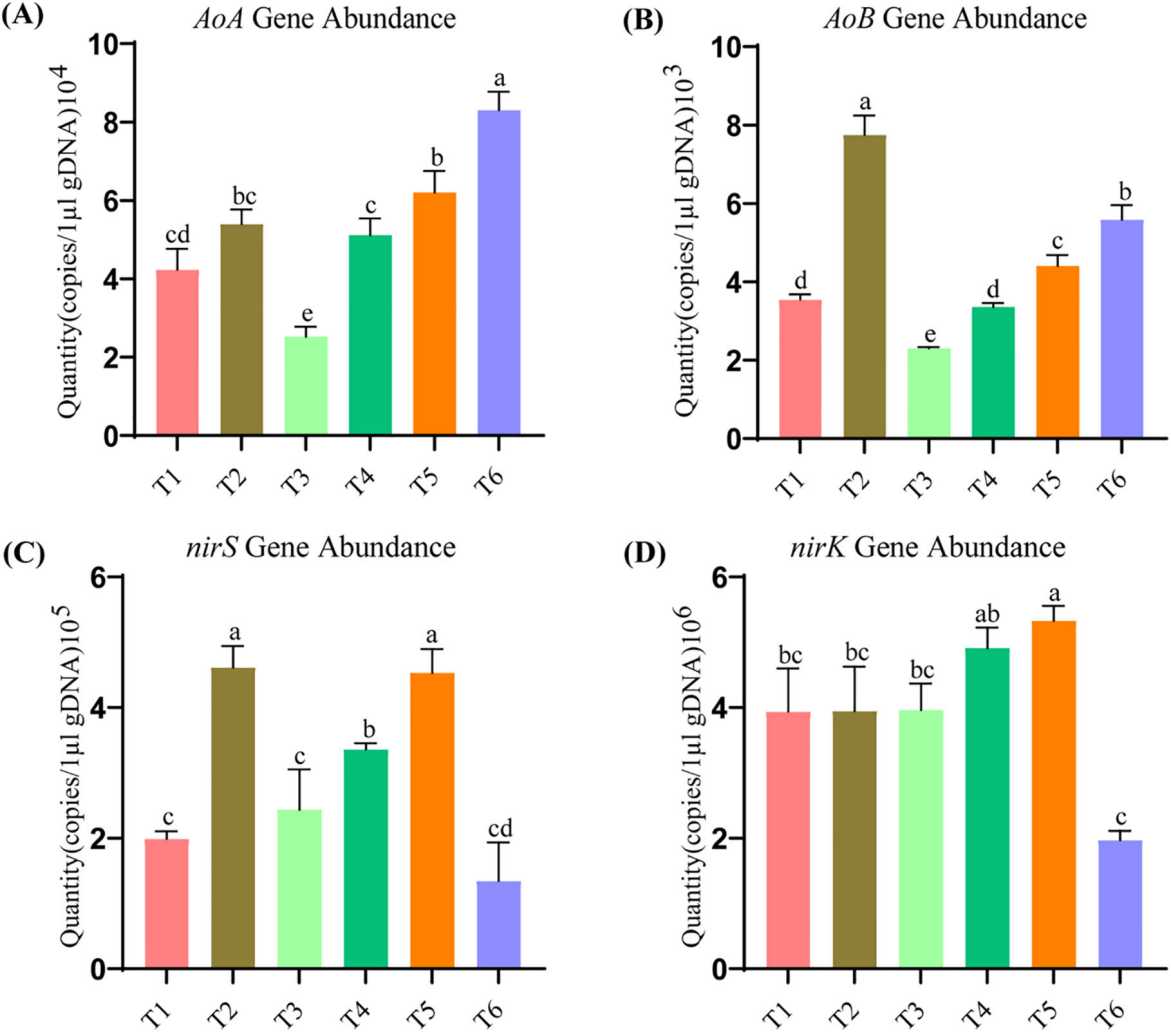
Gene abundance in soil treated with varying levels of organic and inorganic nitrogen fertilization. Ammonia Oxidizing Archaea (*AOA*) **(A)**, Ammonia Oxidizing Bacteria (*AOB*) **(B)**, *nirS***(C)** and *nirK***(D)**. Different letters within the same columns denote statistically significant difference at p>0.05.

**Figure 4A** shows that the abundance of archaeal (*AOA*) gene was significantly higher throughout all treatments, peaking at 8.3 × 1^4^ g d.w.^-1^ in T6 as observed by duplicate analysis. Our results imply that a higher soil organic matter, as regard to plant input and physical protection from degradation of organic matter in this case due to the presence of legacy (carbon/nutrient-rich) additions like manure, may favor archaeal over bacterial ammonia-oxidizing microorganisms. **Figure 4B** reveals that T2 had the highest abundance of bacterial *amoA* (*AOB*) gene at 7.74 × 10³, indicating that mineral inputs may have supported ammonia oxidation by bacteria, followed by abundance in T6, while the combination of fertilizers in T4 and T5 revealed at par abundance, reflecting a potential preference for mixed fertilizer types over pure organic matter for bacterial growth. **Figure 4C** reflects the highest *nirS* gene load with a maximum count of 4.61 × 10^5^ g^-1^ d.w. in T2. This may suggest that the treatments with a higher inorganic nitrogen input induce more an increase of *nirS*-denitrifying bacteria abundance than those characterized by lower industrial fertilization (**Figure 4D**). The *nirK* gene also showed highest at level T5 (at 5.33 × 10^6^ g^-¹^ d.w.). This indicates that *nirK*-denitrifying bacteria are favored in treatments combining organic and synthetic (especially the ones using a greater quantity of organic material) fertilizers. These imply that the sources of nitrogen are different, and both nitrification and denitrification have significant roles, as shown by the order of magnitude of the differences. In other words, the data shows that increasing nitrification and denitrification corresponding to varying sources of nitrogen outlines the broad importance of organic amendments and especially sheep manure in increasing the abundance of ammonia-oxidizing archaea and bacteria as well as denitrifying bacteria. As such, the soils have the potential for nitrogen transformation owing to the fertilization type employed. Indeed the data shows that the type of fertilization played a major role in the prevalence of the genes, subsequently impacting their functional potentials with varying cycle dynamics. Raw data of genes abundance across different organic and inorganic N fertilization treatments are available in the **Supplementary Table 6**.

### 3.8 Pearson correlation coefficient analysis with measured parameters

The Pearson correlation coefficient analysis provides information on the relationships of various soil and plant parameters with the determined microbial and nitrogen cycling variables (**Table 4**). Concerning the NP and RNP with *AOB* abundance, it appeared to be high and equal to *r* = 0.92** and 0.91**, respectively. Accordingly, as nitrification is conducted by NH_3_-oxidizing bacteria, these results prove the high nitrification potential of the soils under the studied fertilization treatments. Moreover, it was also established that NP and RNP were significantly and positively correlated with NO_3_–N (*r* = 0.40 and 0.38, respectively) and grain N (*r* = 0.45 and 0.35, respectively) contents. In such a way, it could be stated that more effective nitrogen cycling contributes to a higher presence of NO_3_–N in soils and its content in plants. Meanwhile, *AOA* abundance did not show strong correlations with the majority of the studied variables, with only one parameter being significantly positively correlated with NH_4_–N (*r* = 0.35). At the same time, it was revealed that *AOB* abundance and DP and RDP were correlated with certain parameters such as *r* = 0.52 and *r* = 0.55*, respectively, being correlated with pH, or with DOC NO_3_–N *r* = 0.48* and 0.60* or 0.50* and 0.65*, respectively, and with leaf N *r* = 0.55* and 0.60*. Another important and highly positively correlated pair was DP with *nirS* and *nirK r* = 0.60* and 0.70*, respectively, or RDP with *nirK r* = 0.75* or *nirS r* = 0.65*. In such a way, it could be considered that DP and RDP highly influence the processes of denitrification, and both *nirS* and *nirK* play a great role in it, while *nirK* might be crucial for the latter. As is totally known, N transformation and certain microbial species are closely linked with soil physicochemical properties, and properly balanced fertilizations are needed to guarantee necessary nutrient content.

**Table 4:** Pearson correlation coefficient analysis with measured parameters.

Variable	pH	EC	DOC	NO_3_-N	NH_4_-N	Grain N	Leaf N	Stem N	NP	RNP	*AOA*	*AOB*	DP	RDP	*nirS*	*nirK*
NP	0.15	0.32	0.35	0.40	0.22	0.45	0.42	0.38	1	0.99**	0.10	0.92**	0.50*	0.54*	0.41	0.48*
RNP	0.12	0.30	0.40	0.38	0.33	0.35	0.38	0.42	0.99**	1	0.12	0.91**	0.55*	0.60*	0.50*	0.55*
*AOA*	-0.25	0.15	0.20	0.30	0.35	0.10	0.12	0.11	0.10	0.12	1	-0.08	0.10	0.11	0.10	0.10
*AOB*	0.55*	0.10	0.15	0.22	0.30	0.15	0.17	0.18	0.92**	0.91**	-0.08	1	0.12	0.13	0.11	0.12
DP	-0.20	0.55*	0.48*	0.60*	0.30	0.48*	0.55*	0.58*	0.50*	0.55*	0.10	0.12	1	0.95**	0.60*	0.70*
RDP	0.18	0.50*	0.50*	0.65*	0.37	0.55*	0.60*	0.62*	0.54*	0.60*	0.11	0.13	0.95**	1	0.65*	0.75**
*nirS*	-0.30	0.52	0.45	0.50*	0.30	0.30	0.32	0.35	0.41	0.50*	0.10	0.11	0.60*	0.65*	1	0.92**
*nirK*	0.48	0.55*	0.50*	0.55*	0.32	0.35	0.40	0.38	0.48*	0.55*	0.10	0.12	0.70*	0.75**	0.92**	1

Values are Pearson correlation coefficients. ** indicates significance at p< 0.01, & * indicates significant at p<0.05.

NP, nitrification potential; RNP, recovered nitrification potential; DP, denitrification potential; RDP, recovered denitrification potential; DOC, dissolved organic carbon.

### 3.9 Heatmap analysis of nitrogen-related gene expression

**Figure 5:**
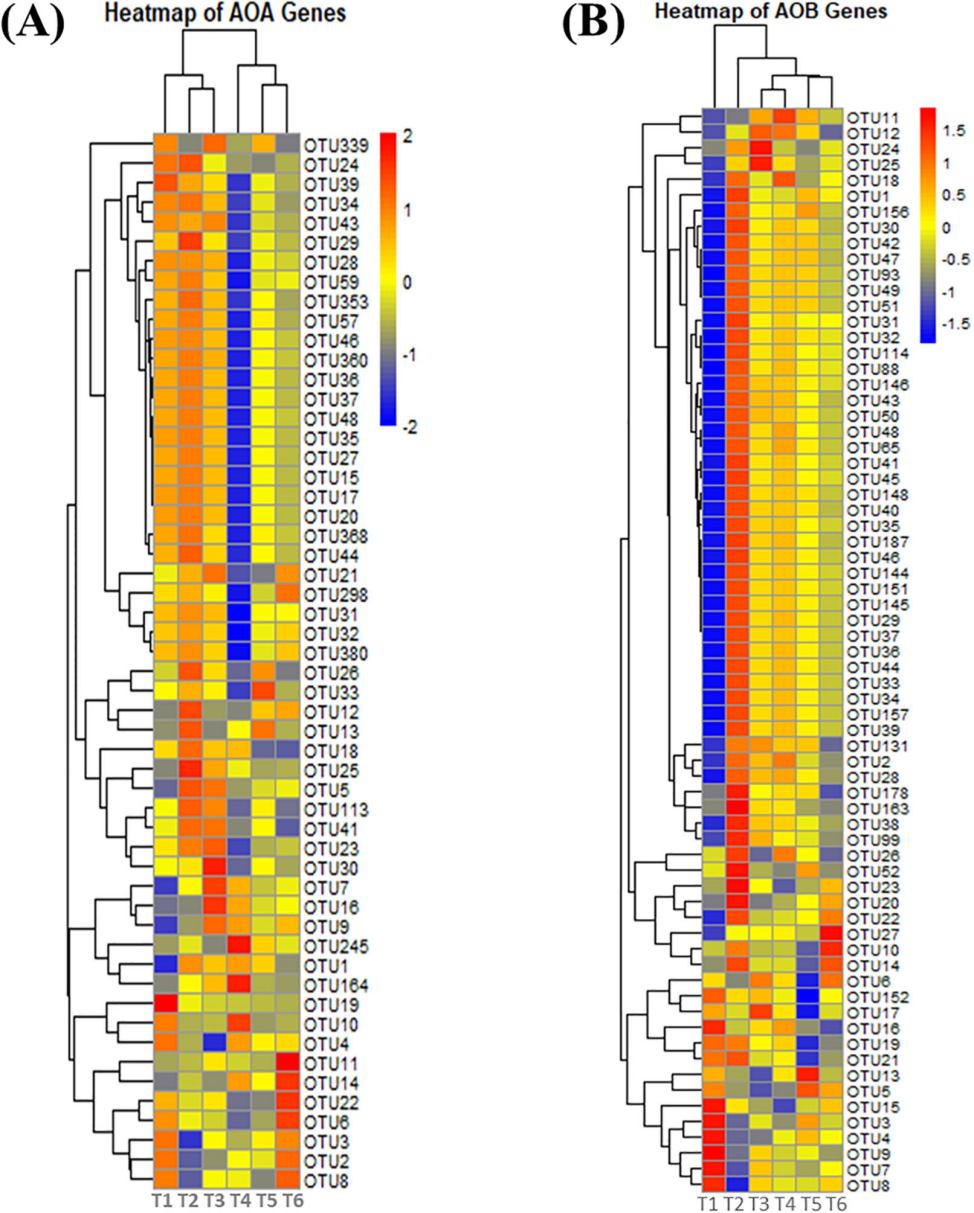
Heatmap of *AOA* and *AOB* gene expression under different levels of organic and inorganic nitrogen fertilization. Ammonia Oxidizing Archaea (*AOA*) **(A)**, Ammonia Oxidizing Bacteria (*AOB*) **(B)**.

The heatmap analyses of gene expression involved in nitrogen cycling based on the four fertilization treatments reveal different responses of *AOA*, *AOB*, *nirK*, and *nirS* genes. Higher expression levels of *AOA* genes (**Figure 5A**, **Supplementary Table 7**) and specific OTUs, including OTU12, OTU13, OTU24, OTU25, OTU26, and OTU29, were found in treatments where inorganic inputs dominated, followed by T3 and a slight expression in T6. This indicates that *AOA* performs better under high-ammonia-availability conditions provided by synthetic fertilizers, which runs counter to the traditional expectation that organic inputs would favor *AOA* activity. As shown in **Figure 5B**, *AOB* gene expression peaks when treatments are applied with inorganic nitrogen inputs (T2) (**Supplementary Table 8**). These treatments are characterized by the dominance of OTUs such as OTU20, OTU22, OTU23, OTU26, and OTU126. The expression follows a decreasing trend as the organic amendments are increasing, suggesting that bacterial ammonia oxidizers are most stimulated in the presence of inorganic nitrogen sources. The results indicate that *AOB* populations thrive best when they are given inorganic nitrogen fertilizers.

**Figure 6:**
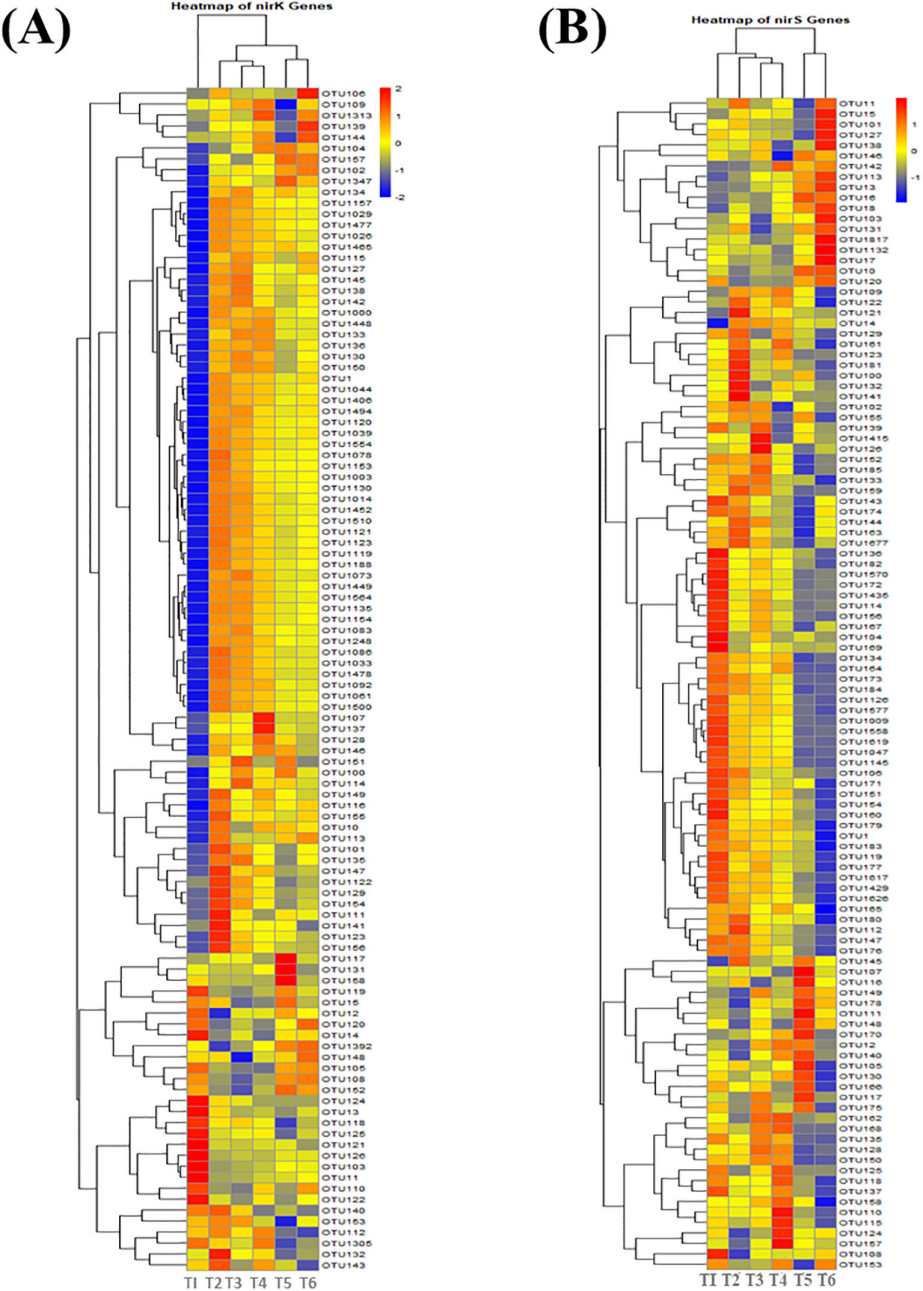
Heatmap of *nirS* and *nirK* gene expression gene expression under different levels of organic and inorganic nitrogen fertilization. Here, *nirK***(A)**, nirS **(B)**.

**Figure 6A**, **Supplementary Table 9** shows that the expression of the *nirK* gene is increased under treatments providing greater organic inputs, including T2 (100% urea) (**Supplementary Table 9**). This indicates that inorganic matter acts as an important source for *nirK*-mediated denitrification under different physiological statuses, such as highly expressed OTUs: OTU101, OTU111, OTU123, OTU147, OTU164, and OTU166. The expression of *nirK*-harboring bacteria was also mediated with the combination of organic and inorganic nitrogen sources, yet their expression become neutral sole organic fertilization. For *nirS* genes, OTUs like OTU11, OTU15, OTU17, OTU101, OTU103, OTU127, OTU132, OTU138, and OTU1817 are overexpressed in the treatments with more organic matter, especially T6 (**Figure 6B**, **Supplementary Table 10**). These genes are essential for denitrification and demonstrate that the conditions created by organic inputs promote microbial denitrification activity. The resulting OTU hierarchical clustering appears to follow distinct grouping patterns, indicating that the microbial community responds differently among nitrogen inputs (organic, inorganic, or combination). The cluster of treatments with high organic inputs suggests that these inputs increase the diversity of microbial communities and/or enhance relevant microbial functions performing nitrogen transformations such as nitrification and denitrification processes. In contrast, organic input dominant treatments tend to cluster separately here due in part to their differential adaptation associated with individual inorganic nitrogen microbial communities.

### 3.10 Principal component analysis of nitrogen-related gene expression

**Figure 7:**
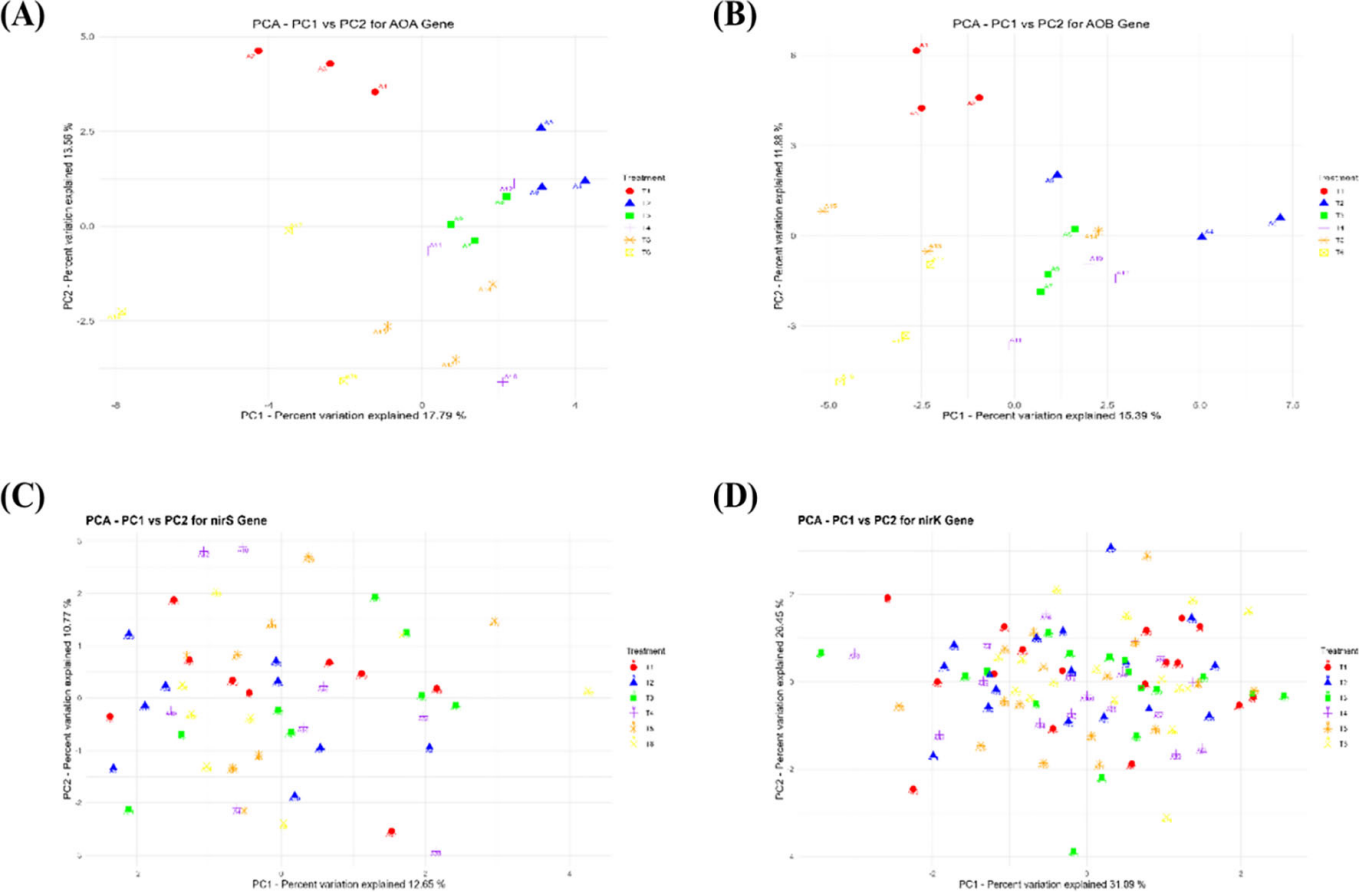
PCA analysis of *AoA*, *AoB*, *nirS*, and *nirK* genes in soil treated with varying levels of organic and inorganic nitrogen Fertilization. Ammonia Oxidizing Archaea (*AOA*) **(A)**, Ammonia Oxidizing Bacteria (*AOB*) **(B)**, *nirS***(C)** and *nirK***(D)**.

Principal component analysis (PCA) indicates the effects of various levels and types of nitrogen treatments on soil N gene expressions. The PCA plot for *AOA* gene expression (**Figure 7A**) shows the delineation of the T6 treatment (high nutrient input) on the far right separately from all other treatments. This implies that *AOA* gene expression is rather sensitive to the organic matter input, which should, in turn, be influenced by environmental parameters stimulating the activity of archaea. On the other hand, the treatments with inorganic nitrogen do not segregate easily according to gene family expression and are generally found to the left of the plot. A distinct cluster on the right side of the figure is formed by T3, T4, and T5 or treatments that received any combination of organic and inorganic inputs (*AOB* gene expression, **Figure 7B**). This demonstrates that the combination of organic and inorganic nitrogen sources promotes the expression of *AOB* genes. The separate clustering seen for T2 (all inorganic) to the left of the ordinations adds further weight to this suggestion and suggests that ammonia-oxidizing bacteria respond quite differently when exposed 100% to inorganic nitrogen: probably due simply to a reduced availability of organic substrates.

For the *nirS* gene expression (**Figure 7C**), the clustering is slightly sharper, with T6 treatments forming their own branch to the right-most side of the plot. The instability in the *nirS* expression under low organic treatments suggests that either these conditions do not sustain viable levels of denitrifying bacteria or that *nirS* gene expression is more specifically reactive to high organic input treatments, which likely correspond with the higher soil DOC and nitrate levels available to denitrifiers. Overlapping clusters for treatments with mixed inputs (T3, T4, and T5) indicate that these treatments balance conditions suitable for denitrification, whereas the one uniquely located T2 (inorganic only) on the left shows its relatively lesser contribution to this phenomenon. The *nirK* gene expression (**Figure 7D**) clusters were more dispersed with a great deal of overlap among treatments, most notably at the center of the plot. Given the abovementioned facts, we then go on to assume that the *nirK* gene expression does not respond in a different scale when subjected to nitrogen source types. Their placement also had a slight shift to the right associated with the clustering of treatments with higher organic inputs (T6), suggesting some residual influence of organic matter.

### 3.11 Redundancy analysis of nitrogen-related gene expression

**Figure 8:**
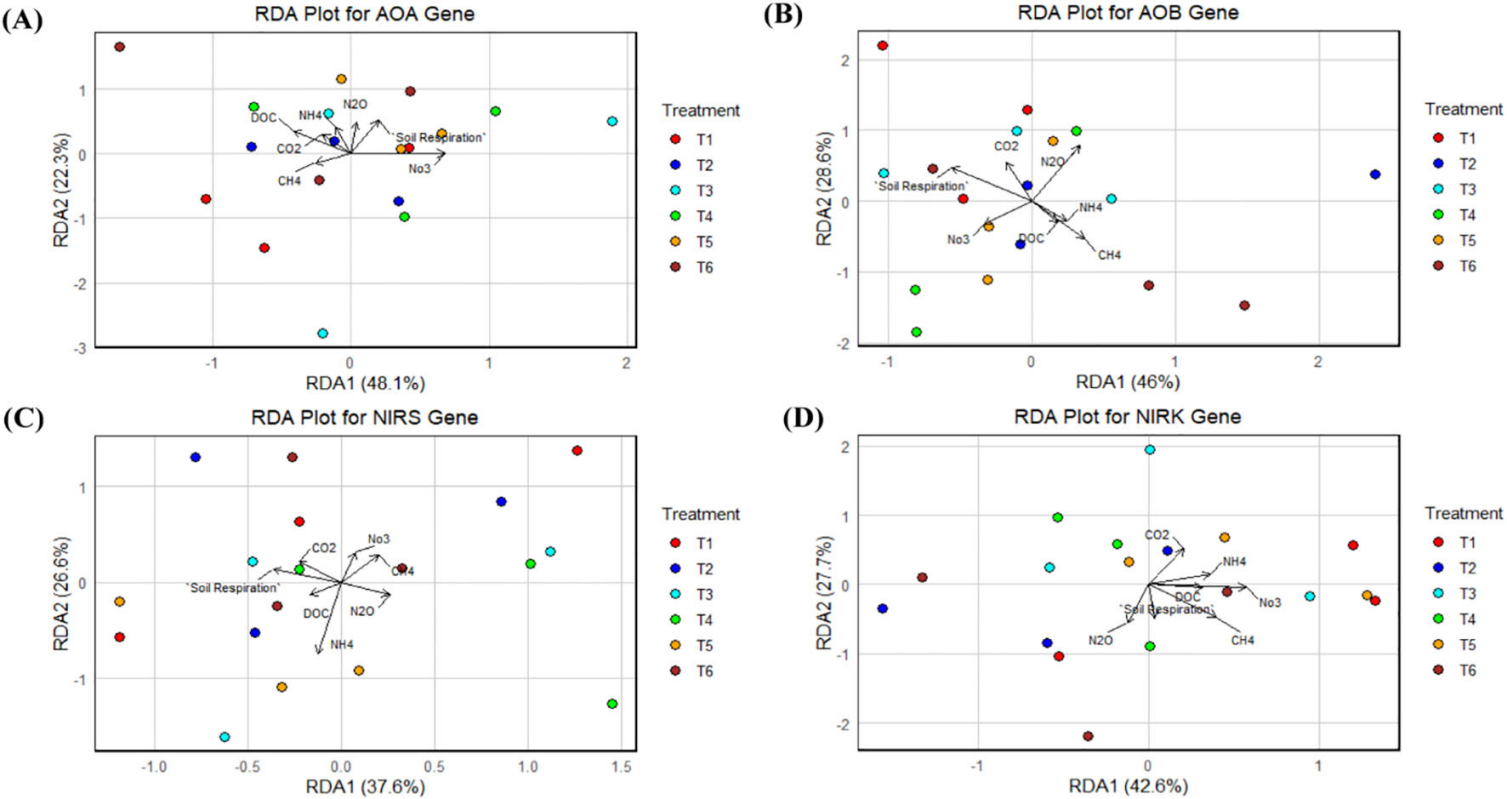
RDA Analysis of *AOA*, *AOB*, *nirS* and *nirK* genes in soil treated with varying levels of organic and inorganic nitrogen fertilization. Ammonia Oxidizing Archaea (*AOA*) **(A)**, Ammonia Oxidizing Bacteria (*AOB*) **(B)**, *nirS***(C)** and *nirK***(D)**.

The RDA analyses demonstrate the relationships between environmental variables and gene expression profiles for the *AOA*, *AOB*, *nirK*, and *nirS* genes across different fertilization treatments. The first two axes from RDA which correspond to *AOA* gene expressions (**Figure 8A**) are displayed in the RDA plot, explaining 48.1% and 22.3% of variance across metagenomes, respectively, meaning that a reasonable proportion of gene expression variance was explained by these axes. However, the vectors for DOC, NH_4_^+^, and N_2_O and soil respiration are strongly associated with *AOA* gene expression in T6 (100% sheep manure) and T4 (30% sheep manure). *AOA* inputs and NH_4_ and availability were, hence, influential in determining the expression of *AOA* genes, amplifying the nitrification potential in these treatments. **Figure 8B** shows that the modeled RDA1 and RDA2 explains 46% and 28.6% of the variation in *AOB* gene expression, respectively. The vectors of DOC, CO_2_, NO_3−_, and NH_4_^+^ are also close between treatments T3, T4, and T5 (a case in which both organic and inorganic materials were applied). This would indicate that there is a complementary role of inorganic and organic nitrogen for the expression of *AOB* functional gene, in particular for ammonium and nitrate together. The T2 treatment (inorganic only) is clustered to the right of this quadrant, indicating that its response under synthetic nitrogen fertilization differed, presumably due to an absence of organic matter input.

For denitrification genes, **Figure 8C** exploring RDA1 explains 37.6% variance for the *nirS* gene expression, while RDA2 accounts for 26.6%. For the NH_4_^+^, DOC, and N_2_O vectors, their high contributions were strongly related to T6 (100% sheep manure) and T5 (45% sheep manure), where organic nitrogen inputs stimulated by ammonium availability dominate *nirS* gene expression. It is indicative that the organic amendment stimulates denitrification, lowering the nitrate contents and enhancing nitrogen gas emissions. **Figure 8D** shows the RDA plots of the *nirK* gene expression which revealed that RDA1 and RDA2 only explained 42.6% and 27.7% of the variation. This figure emphasizes a significant ring of DOC, soil respiration, and N_2_O in *nirK* gene expression control, especially for the treatments with mixed or organic inputs, i.e., T3, T4, T5, and T6. This suggests that in the denitrification processes, organic carbon stimulates the expression of the *nirK* gene and acts as an electron donor.

### 3.12 Alpha diversity indices’ values across different fertilization treatments

The patterns of microbial community structure based on the alpha diversity indices across different nitrogen fertilization treatments are discussed as follows. As shown in **Supplementary Table 2**, the analysis of the *AOA* gene indicates that the diversity indices for different treatments present significant differences. Specifically, T2, corresponding to inorganic fertilizer, has the highest Shannon and Simpson indices at 2.631 ± 0.06 and 0.893 ± 0.003, respectively, indicative of higher diversity and evenness compared to other treatments. T4, relating to 30% sheep manure, has the highest Chao1, and the values in the lower index are lower for T6 or 100% sheep manure. **Supplementary Table 3** illustrates the *AOB* gene results, showing that the trends are similar and that T2 again has the highest Shannon and Simpson indices. The Chao1 index is also higher for T4 and lower for T6. **Supplementary Table 4**, illustrating the *nirS* gene data, shows that the coverage for all treatments is similar, while T5 or 45% sheep manure has the highest Shannon and Simpson indices. T1’s values for the diversity indices are the lowest, similar to the *AOA* and *AOB* gene samples. As for the *nirK* gene, the results in **Supplementary Table 5** suggest that diversity is highest for T6, and the values in the Shannon and Simpson indices are 2.037 ± 0.06 and 0.600 ± 0.010, respectively. The highest Chao1 index is indicated in **Supplementary Table 5** for T4, and the values in the lower index are lowest for T1. Thus, the data in these tables show that different nitrogen sources significantly impact the diversity, richness, and evenness of microbial communities. In addition, these results indicate that the inorganic and combined treatments generally demonstrate a higher diversity than the organic treatments only.

## 4 Discussions

### 4.1 Soil and plant parameters change under different fertilization treatments

The results of the study clearly show significant differences in soil and plant parameters depending on the type of nitrogen fertilization (see **Table 2**). Firstly, the application of 100% sheep manure resulted in significantly higher concentrations of soil DOC and NO_3_–N compared to the rest of the treatments (Yu et al., 2024). It can be assumed that the inputs of organic matter enhance the activity of microbes, which, in turn, allows for a more intensive process of nitrogen mineralization and, subsequently, nitrification (Wang et al., 2023; Liu et al., 2025). Moreover, higher levels of DOC in T6 indicate higher levels of readily available carbon, stimulating a more diverse and, consequently, more active microbial community, responsible for nutrient cycling and other aspects of soil activity (Lazcano et al., 2013). Soil respiration was also significantly higher in T6, indicating enhanced microbial activity and organic matter decomposition. T2, in its turn, was characterized by the highest content of grain N, which can be explained by the *de facto* capacity of inorganic fertilization to provide nitrogen in a form that is immediately available for plants and therefore soil N content. Nevertheless, it is likely that the regular use of only inorganic fertilizers may lead to poor soil structure and fertility in the long run. It must be concluded that both organic and inorganic fertilization should be used in conjunction to ensure the optimized content of immediately available nutrients as well as to promote long-term soil health (Zhou et al., [[NoYear]]; Chen et al., 2024).

### 4.2 Nitrification potential and microbial community dynamics

From the measurement of nitrification potential and recovered nitrification potential, it is evident that the highest nitrification activity was observed in T2 inorganic fertilizer than the high organic input treatment, T6. Nitrification provided more ammonia in T2 than in other treatments. This is because inorganic fertilization has provided an immediate burden for the nitrification process. This is because the direct availability of inorganic nitrogen form permitted both *AOA* and *AOB* to cope up, boosting nitrification (Ouyang and Norton, 2020; Langenfeld, 2025). T6, even though it did not exhibit the highest nitrification potential, provided a suitable home for the fraction of this microorganism, particularly *AOA*, and responded to this treatment. *AOA* and *AOB* exhibited differences in treating organic and inorganic input and showed that they occupy different ecological environments. There existed a diverse size of *AOA* and *AOB* across the treatments, showing that a wide size of this microorganism is responsible for the nitrification carrying capacity of the soil. They are crucial in nitrification, maintaining soil health, and stabilizing soil nitrogen and mineralization of nitrogen (Raglin et al., 2022). Combined organic and inorganic fertilization offers the highest nitrogen recovery, optimizing both immediate crop uptake and long-term soil retention. In contrast, sole inorganic fertilization provides moderate recovery, while organic fertilization enhances soil retention but delivers a lower immediate uptake (Shi et al., 2024).

### 4.3 Denitrification potential and functional gene expression

DP was the highest in T6, where a large amount of organic materials was given. This can be explained by the fact that organic matter is an important factor acting as an electron donor in the process of denitrification. The two types of denitrifiers, namely, *nirS* and *nirK*, were also highly represented in this group, which implies that a big number of microorganisms capable of this reduction ability was developed to depress the levels of nitrate accidents and leaching hazards (Hernández Maqueda et al., 2024; Maqueda et al., 2024). Organic and combined nitrogen fertilization, respectively, enhance denitrification potential and recovery more effectively than inorganic fertilizers alone, largely by increasing the abundance and activity of *nirS*- and *nirK*-type denitrifiers. *nirS* denitrifiers are especially important for denitrification potential, while *nirK* denitrifiers are more sensitive to fertilizer type and soil conditions. These dynamics are crucial to manage soil nitrogen cycling and mitigate N_2_O emissions in agriculture (Shakywal et al., 2023). These results mean that nitrification can be influenced or shaped by the input of organic amendments, which also reduces the greenhouse effect because one of the main byproducts of nitrate utilization is N_2_O, which is considered a potent greenhouse gas. Overall, the study has proved that these two processes always existing in nature can be highly affected depending on the selective input of N-contiguous materials, and they are not always automatically correlated with the amount of urea also added (Li et al., 2022; Han et al., 2024).

### 4.4 Emissions and soil quality under different fertilization treatments

The study shows that different fertilization treatments have their impacts on greenhouse gas emissions and soil quality, as shown in **Supplementary Tables 1**, **2**. For N_2_O emissions, the inorganic fertilizers made the largest contribution, demonstrating its risks of environmental pollution. For T6, N_2_O emissions are the lowest, while the emissions of CO_2_ and CH_4_ are the highest. In this case, it may be assumed that the presence of sheep manure superior to other fertilizers increases the activity of microorganisms that decompose organic substances, liberating gases such as CO_2_ and CH_4_. In addition, CO_2_ is produced by decomposition of the organic contents of the soil (Zheng et al., 2019; Zhang et al., 2024). As the N_2_O emissions do not always correlate with soil quality, which is almost similar across all of the treatments, the main indicator of the latter is organic matter, which is present in greatest quantities in T6. Thus, it may be concluded that in order to increase the sustainability of agroecosystems and soil quality, a balanced type of fertilization should be used (Galic et al., 2019; Lazcano et al., 2021).

### 4.5 Microbial community dynamics and soil health

Soil microbial community structure and function are significantly altered by the integration of nitrogen strategies. This conclusion is underpinned by the heatmap analysis, PCA, and RDA outcomes. The heatmap analysis justified that the *AOA*, *AOB*, *nirK*, and *nirS* gene expressions are different based on treatments. Moreover, it is clear that the organic forms of the amendments, especially sheep manure, might increase the number and activity of these microbes (Wang et al., 2023). It is as a consequence of the presence of organic carbon that acts as an energy source and encourages diverse and active communities. PCA allows for the further decomposition of the modification effect on the compositions of the soil microbes. In this case, both RDA1 and RDA2 (i.e., the two most important components) separate clearly the treatments, including especially T6 (organic) and T2 (inorganic). This clustering can be observed in the PCA plots of *AOA* and *AOB* gene expression, clearly illustrating the separation between organic inputs (T6) and inorganic-only treatments (T2), with a further gradient unrelated to N treatment following for both archaeal nitrification enzymes. Such an outcome confirmed the possibility of the development of unique niches within the soils and supports differential microbial activity and capabilities, especially between ammonia-oxidizing archaea and bacteria (Dincă et al., 2022). The distance with which the *nirK* and *nirS* gene expression clusters resolved in PCA plots highlights this varying response of denitrifiers to different nitrogen sources, with organic inputs leading to highest activity (Xiao X. et al., 2021).

Finally, the results of the RDA showed a strong connection between environmental factors, like DOC, NO_3_, NH_4_, N_2_O, CO_2_, CH_4_, and soil respiration, and their effect on gene expression. The case with nitrate levels and the expression of nitrification and denitrification operations was an excellent example of such interrelationship (Ge et al., 2010). In particular, DOC and NH_4_^+^ were positively associated with greater *AOA* and *AOB* gene expression in mechanically disaggregated organic-rich treatments, while N_2_O and CO_2_ emissions corresponded to denitrification activities in all organically amended or unamended mixed (T3, T4, T5) treatments as well as an organic-enriched treatment (T6). The compelling effects of environmental factors (e.g., pH, moisture, and nitrate/ammonium) on gene expression are corroborated by the RDA results as well. Denitrification processes catalyzed by *nirK* and *nirS* genes were significantly sensitive to DOC and N_2_O levels especially under high-organic-nitrogen inputs. Nitrification was predominantly driven by nitrate, and the main control on denitrification varied between moisture availability for T6 (high organic inputs) and ammonium for treatments receiving equivalent amounts of N as NH_4_ and NO_3_ (Kamaa et al., 2011).

This interaction highlights the importance of fertilization options that are able to maintain a good level of microbial diversity and function on soils. Simultaneous supplementation of organic and inorganic nitrogen inputs improves the balance of nutrients without harming the nutrient turnover rate, enhances microbial activity, and decreases negative effects on the environment, like nitrate leaching or greenhouse gas emissions. This demonstrates the need for a combined approach to ensure good nitrogen cycling and minimum impact on science health from both organic inputs as well as inorganic inputs (Ling et al., 2017; Pantelides et al., 2023; Kumar et al., 2025).

### 4.6 For agricultural practices and environmental sustainability

The results of the study contain strong evidence that the combined use of organic and inorganic nitrogen fertilization has multiple benefits. While inorganic fertilizers facilitate the immediate availability of nutrients, it does not support the long-term health of soil or microbial diversity (Bilalis et al., 2018; Shi et al., 2024). In its turn, organic additions, such as sheep manure, improve the structure of the soil, increase the microbial diversity, and supports such processes as both nitrification and denitrification (Thangarajan et al., 2013; Goldan et al., 2023). Our findings are consistent with the broader literature on integrated nutrient management. Specifically, the optimal 30% substitution rate of organic manure identified here corroborates the work of (Liu et al., 2021; Wang et al., 2025), who found that moderate organic integration best synchronizes nitrogen release with crop demand. This is likely to mean that the use of a combination of the mechanisms allows for the optimization of the processes of soil nutrient cycling and management, supports improved nutrient uptake processes of the plants, and minimizes the dangers of nitrogen leaching and gaseous emissions. This is an important finding for the development of environmentally sustainable practices.

## 5 Conclusion

Based on the observations in this study, it is clear that the different nitrogen fertilization treatments had a considerable impact on soil and plant parameters, GHG emissions, and the soil-associated microbial community. The application of sheep manure significantly increased soil-dissolved organic carbon and nitrate levels, enhancing the operation of a more diverse and active microbial community. This is further corroborated by the increase in the abundance of *AOA* and optimization of *AOB* gene expressions under organic treatments. On the other hand, organic matter plays a vital role in boosting microbial processes, as indicated by the increase in the rates of nitrification and denitrification in the current study. In contrast, inorganic fertilization provides a readily available nitrogen source, leading to the highest grain N content. Nonetheless, it exhibits a shortfall of the continuous introduction of organic matter that sustains soil’s long-term fertility. The current study concludes that integrating inorganic and organic sources for optimal synchrony, an integrated 30% organic with 70% inorganic application of N, will significantly improve the nutrient availability in soils without perceptibly compromising the nature of soil structure or the diversity of the soil microbial community. Therefore, there is a rising need to cautiously apply inorganic fertilizers to the soil when the subsequent test indicates a deficiency in the nutrients to achieve proper fertilization.

## Data Availability

The original contributions presented in the study are publicly available. This data can be found here: NCBI, *AOA*: PRJNA1366377, *AOB*: PRJNA1366415, *nirS*: PRJNA1366420, *nirK*: PRJNA1366424.

## Supplementary material

Supplementary Table 1 Implementation Plan for Sheep Manure as Synthetic Fertilizer Replacement (2018-2019).

Supplementary Table 2 Alpha Diversity Indices Values for AOA Gene.

Supplementary Table 3 Alpha Diversity Indices Values for AOB Gene.

Supplementary Table 4 Alpha Diversity Indices Values for nirS Gene.

Supplementary Table 5 Alpha Diversity Indices Values for nirK Gene.

Supplementary Table 6 Gene abundance data.

Supplementary Table 7 AOA structure.

Supplementary Table 8 AOB structure.

Supplementary Table 9 nirK structure.

Supplementary Table 10 nirS structure.
