# Supplementary material for: Integration of organic–inorganic nitrogen fertilization on nitrogen conversion in soil
Source: Front Plant Sci. 2025 Dec 10;16:1688878. doi: 10.3389/fpls.2025.1688878 (PMC12728020; doi:10.3389/fpls.2025.1688878)
Supplement: Supplementary Table 1 — Implementation Plan for Sheep Manure as Synthetic Fertilizer Replacement (2018-2019). [file Table1.docx]

**Supplementary Table 1.** Implementation Plan for Sheep Manure as Synthetic Fertilizer Replacement (2018-2019)

|  | **Sheep manure fertilizer (kg·hm^-2^ )** | | |  |  |  | **Chemical fertilizer (kg·hm^-2^ )** | |
| --- | --- | --- | --- | --- | --- | --- | --- | --- |
| **Treatments** | **Fertilizer** |  |  |  |  |  |  |  |
|  | **Quantity** | **N** | **P** | **K** |  | **N** | **P** | **K** |
|  |  |  |  |  |  |  |  |  |
| **T1** | 0.00 | 0.00 | 0.00 | 0.00 |  | 0.00 | 0.00 | 0.00 |
| **T2** | 0.00 | 0.00 | 0.00 | 0.00 |  | 0.95 | 1.57 | 2.88 |
| **T3** | 22.27 | 0.22 | 0.12 | 0.35 |  | 0.58 | 1.30 | 2.18 |
| **T4** | 44.54 | 0.43 | 0.24 | 0.70 |  | 0.22 | 1.04 | 1.48 |
| **T5** | 66.82 | 0.65 | 0.36 | 1.05 |  | 0.16 | 2.54 | 0.78 |
| **T6** | 148.48 | 1.44 | 0.891 | 2.33 |  | 0.00 | 0.00 | 0.00 |
