# Supplementary material for: Integration of organic–inorganic nitrogen fertilization on nitrogen conversion in soil
Source: Front Plant Sci. 2025 Dec 10;16:1688878. doi: 10.3389/fpls.2025.1688878 (PMC12728020; doi:10.3389/fpls.2025.1688878)
Supplement: Supplementary Table 2 — Alpha Diversity Indices Values for AOA Gene. [file Table2.docx]

**Supplementary Table 2.** Alpha Diversity Indices Values for AOA Gene

| **Treatment** | **Coverage** | **Chao1** | **Shannon** | **Pielou** | **Simpson** |
| --- | --- | --- | --- | --- | --- |
| T1 | 0.164703 ± 0.001 | 55.25 ± 1.00 | 2.373 ± 0.05 | 0.592 ± 0.01 | 0.857 ± 0.002 |
| T2 | 0.153280 ± 0.002 | 54.00 ± 1.20 | 2.631 ± 0.06 | 0.660 ± 0.02 | 0.893 ± 0.003 |
| T3 | 0.167471 ± 0.001 | 52.00 ± 1.30 | 2.381 ± 0.04 | 0.603 ± 0.01 | 0.853 ± 0.003 |
| T4 | 0.165389 ± 0.002 | 58.50 ± 1.50 | 2.472 ± 0.05 | 0.609 ± 0.01 | 0.869 ± 0.002 |
| T5 | 0.166896 ± 0.001 | 55.00 ± 1.10 | 2.360 ± 0.04 | 0.592 ± 0.01 | 0.848 ± 0.002 |
| T6 | 0.182261 ± 0.003 | 53.00 ± 1.20 | 2.244 ± 0.03 | 0.565 ± 0.01 | 0.821 ± 0.003 |
