# Supplementary material for: Integration of organic–inorganic nitrogen fertilization on nitrogen conversion in soil
Source: Front Plant Sci. 2025 Dec 10;16:1688878. doi: 10.3389/fpls.2025.1688878 (PMC12728020; doi:10.3389/fpls.2025.1688878)
Supplement: Supplementary Table 3 — Alpha Diversity Indices Values for AOB Gene. [file Table3.docx]

**Supplementary Table 3.** Alpha Diversity Indices Values for AOB Gene

| **Treatment** | **Coverage** | **Chao1** | **Shannon** | **Pielou** | **Simpson** |
| --- | --- | --- | --- | --- | --- |
| T1 | 0.058065 ± 0.001 | 48.92 ± 1.50 | 2.449 ± 0.05 | 0.630 ± 0.01 | 0.871 ± 0.003 |
| T2 | 0.056344 ± 0.001 | 50.33 ± 1.60 | 2.515 ± 0.06 | 0.645 ± 0.01 | 0.874 ± 0.002 |
| T3 | 0.056825 ± 0.002 | 50.33 ± 1.40 | 2.421 ± 0.04 | 0.623 ± 0.01 | 0.862 ± 0.002 |
| T4 | 0.058847 ± 0.002 | 52.25 ± 1.70 | 2.409 ± 0.05 | 0.612 ± 0.01 | 0.858 ± 0.003 |
| T5 | 0.061494 ± 0.002 | 45.50 ± 1.20 | 2.297 ± 0.04 | 0.607 ± 0.01 | 0.836 ± 0.003 |
| T6 | 0.062637 ± 0.002 | 47.71 ± 1.30 | 2.236 ± 0.05 | 0.579 ± 0.01 | 0.821 ± 0.002 |
