# Supplementary material for: Integration of organic–inorganic nitrogen fertilization on nitrogen conversion in soil
Source: Front Plant Sci. 2025 Dec 10;16:1688878. doi: 10.3389/fpls.2025.1688878 (PMC12728020; doi:10.3389/fpls.2025.1688878)
Supplement: Supplementary Table 4 — Alpha Diversity Indices Values for nirS Gene. [file Table4.docx]

**Supplementary Table 4.** Alpha Diversity Indices Values for nirS Gene

| **Treatment** | **Coverage** | **Chao1** | **Shannon** | **Pielou** | **Simpson** |
| --- | --- | --- | --- | --- | --- |
| T1 | 0.162879 ± 0.003 | 109.00 ± 3.00 | 3.097 ± 0.05 | 0.660 ± 0.01 | 0.873 ± 0.008 |
| T2 | 0.162875 ± 0.003 | 112.25 ± 3.50 | 3.363 ± 0.06 | 0.713 ± 0.01 | 0.906 ± 0.009 |
| T3 | 0.164204 ± 0.004 | 110.00 ± 3.20 | 3.362 ± 0.06 | 0.715 ± 0.02 | 0.908 ± 0.009 |
| T4 | 0.179202 ± 0.004 | 112.00 ± 3.50 | 3.422 ± 0.06 | 0.725 ± 0.02 | 0.915 ± 0.010 |
| T5 | 0.155209 ± 0.002 | 112.00 ± 3.00 | 3.591 ± 0.07 | 0.761 ± 0.02 | 0.935 ± 0.012 |
| T6 | 0.175631 ± 0.004 | 112.00 ± 3.40 | 3.522 ± 0.07 | 0.747 ± 0.02 | 0.937 ± 0.011 |
