# Supplementary material for: Integration of organic–inorganic nitrogen fertilization on nitrogen conversion in soil
Source: Front Plant Sci. 2025 Dec 10;16:1688878. doi: 10.3389/fpls.2025.1688878 (PMC12728020; doi:10.3389/fpls.2025.1688878)
Supplement: Supplementary Table 5 — Alpha Diversity Indices Values for nirK Gene. [file Table5.docx]

**Supplementary Table 5.** Alpha Diversity Indices Values for nirK Gene

| **Treatment** | **Coverage** | **Chao1** | **Shannon** | **Pielou** | **Simpson** |
| --- | --- | --- | --- | --- | --- |
| T1 | 0.200453 ± 0.003 | 93.50 ± 2.00 | 0.936 ± 0.02 | 0.207 ± 0.01 | 0.261 ± 0.005 |
| T2 | 0.168013 ± 0.002 | 99.50 ± 2.50 | 1.056 ± 0.03 | 0.230 ± 0.01 | 0.305 ± 0.006 |
| T3 | 0.170401 ± 0.002 | 107.00 ± 3.00 | 1.268 ± 0.04 | 0.272 ± 0.01 | 0.365 ± 0.007 |
| T4 | 0.166708 ± 0.003 | 116.00 ± 2.50 | 1.469 ± 0.05 | 0.314 ± 0.02 | 0.432 ± 0.008 |
| T5 | 0.160429 ± 0.002 | 105.17 ± 2.00 | 1.431 ± 0.04 | 0.307 ± 0.01 | 0.414 ± 0.007 |
| T6 | 0.133997 ± 0.002 | 110.50 ± 2.80 | 2.037 ± 0.06 | 0.437 ± 0.02 | 0.600 ± 0.010 |
